# VasH Contributes to Virulence of *Aeromonas hydrophila* and Is Necessary to the T6SS-mediated Bactericidal Effect

**DOI:** 10.3389/fvets.2021.793458

**Published:** 2021-12-13

**Authors:** Jihong Li, Zhihao Wu, Changsong Wu, Dan-Dan Chen, Yang Zhou, Yong-An Zhang

**Affiliations:** ^1^Institute of Hydrobiology, Chinese Academy of Sciences (CAS), Wuhan, China; ^2^University of Chinese Academy of Sciences, Beijing, China; ^3^State Key Laboratory of Agricultural Microbiology, College of Fisheries, Huazhong Agricultural University, Wuhan, China; ^4^Engineering Research Center of Green Development for Conventional Aquatic Biological Industry in the Yangtze River Economic Belt, Ministry of Education, Wuhan, China; ^5^Guangdong Laboratory for Lingnan Modern Agriculture, Guangzhou, China

**Keywords:** *Aeromonas hydrophila*, whole-genome sequencing, T6SS, VasH, virulence

## Abstract

*Aeromonas hydrophila* is a Gram-negative bacterium that is commonly distributed in aquatic surroundings and has been considered as a pathogen of fish, amphibians, reptiles, and mammals. In this study, a virulent strain *A. hydrophila* GD18, isolated from grass carp (*Ctenopharyngodon idella*), was characterized to belong to a new sequence type ST656. Whole-genome sequencing and phylogenetic analysis showed that GD18 was closer to environmental isolates, however distantly away from the epidemic ST251 clonal group. The type VI secretion system (T6SS) was known to target both eukaryotic and prokaryotic cells by delivering various effector proteins in diverse niches by Gram-negative bacteria. Genome-wide searching and hemolysin co-regulated protein (Hcp) expression test showed that GD18 possessed a functional T6SS and is conditionally regulated. Further analysis revealed that VasH, a σ54-transcriptional activator, was strictly required for the functionality of T6SS in *A. hydrophila* GD18. Mutation of *vas*H gene by homologous recombination significantly abolished the bactericidal property. Then the virulence contribution of VasH was characterized in both *in vitro* and *in vivo* models. The results supported that VasH not only contributed to the bacterial cytotoxicity and resistance against host immune cleaning, but also was required for virulence and systemic dissemination of *A. hydrophila* GD18. Taken together, these findings provide a perspective for understanding the VasH-mediated regulation mechanism and T6SS-mediated virulence and bactericidal effect of *A. hydrophila*.

## Introduction

*Aeromonas hydrophila* is an opportunistic pathogen widespread in aquatic environments. This bacterium could cause multiple diseases in different animal species, such as fish, amphibians, reptiles, and humans (1). In fish, *A. hydrophila* can cause outbreaks of motile *Aeromonas* septicemia (MAS) with symptoms including reddened fins, inflammation of the anus, diffuse hemorrhages on the skin, exophthalmia, and abdominal swelling (2). This pathogen has frequently caused a high mortality rate in commercial aquaculture throughout China since 1989 (3). In recent years, MAS caused by *A. hydrophila* has hindered the rapid development of carp industry in China and catfish industry in the United States (4, 5). Grass carp (*Ctenopharyngodon idellus*) is the fish species with the most significant reported production in aquaculture globally, with a proportion of up to 5.5 million tons per year (6). Increased incidence of infection and the broad spectrum of antibiotic resistance has made *A. hydrophila* a severe threat to the aquaculture industry.

The dynamic characteristics and overlapping classification have made the turbulent nature of classification within *Aeromonas* spp. (7). Multilocus sequence typing (MLST) permitted accurate strain genotyping and the phylogenetic evaluation of concatenated core genome gene sequences, offering a valuable tool for epidemiological outbreak tracing, host range evolution, and ecological research (8). The derived sequence types (STs) shed light on the relationship among the taxa belonging to the genus *Aeromonas*. ST251 is regarded as the virulent strain clonal group of *A. hydrophila*, accountable for the recent years' MAS (9). However, MLST, defined through housekeeping genes as sequence types (STs) and clone groups, has limited ability to further identify genetically related strains in STs. Latest, whole-genome sequencing (WGS) of pathogens has become more accessible and affordable as a tool for regular monitoring and detection of a potential outbreak. It offers information on the bacterial genome at a much more satisfactory resolution than MLST (10). Application of WGS made accurate diagnoses possible and has facilitated the investigations of disease outbreaks (11). Although there have been increasing *A. hydrophila* genome sequences available in the database, complete whole genome sequence and detailed genomic analysis of grass carp isolated strains are still very limited.

The Type VI Secretion System (T6SS) is a versatile weapon employed by bacteria to protect themselves against predators, disrupt eukaryotic cells, and fight against different microorganisms (12). As identified in more than 25% of sequenced Gram-negative bacteria, T6SS is a contact-dependent toxin delivery machine that can directly kill competitors or hosts through protein toxin translocation (13–16). The component of T6SS has 13 core genes, while additional elements likely to participate in the delivery of the effector (17). The tail tube of T6SS is made of hemolysin co-regulated protein (Hcp), capped by a puncturing device containing proteins (12). Hcp is essential for the structural integrity of T6SS apparatus and Hcp could be secreted with different effectors (18, 19). The secretion of Hcp is a dependable marker of workable T6SS (20). T6SSs are strictly regulated and the transcription was directly or indirectly modified by regulators, including the QS system, sigma 54 factors, H-NS, and Fur (17, 21–23). In *Vibrio cholerae* and *V. fischeri*, VasH is a transcriptional regulator of T6SS and contains a DNA-binding sigma54 motif, which is critical for the ability to activate transcription of T6SS genes (24–26). Earlier studies showed that the deletion of *vas*H inhibited the expression and secretion of Hcp in *A. dhakensis* SSU, previously considered as an *A. hydrophila* strain (7, 23). In *A. hydrophila*, many of the T6SS components still await demonstration of function, including whether VasH is deployed and the role it plays in *A. hydrophila* survival and infection.

In the present study, an *A. hydrophila* strain GD18 was isolated from diseased grass carp. MLST analysis showed that GD18 belongs to a new sequence type ST656. To further discriminate GD18 genetically, WGS was applied and the evolution relationship between GD18 and other *A. hydrophila* isolates was clarified. Further analysis found that *A. hydrophila* GD18 possesses a complete and functional T6SS. Then the role of VasH in T6SS-mediated virulence and the bactericidal effect was preliminarily deciphered.

## Materials and Methods

### Plasmids, Bacterial Strains, Cell Line, and Experimental Fish

For the bacterial strains, plasmids, and primers used in this study, see Table 1 and Supplementary Table 1. *A. hydrophila* strain GD18 was isolated from diseased grass carp (*C. idella*). The morphology of bacterial cells was determined by transmission electron microscopy (TEM; Hitachi H-7650, Japan). The β-hemolytic phenotype was observed on sheep's blood agar. Luria Agar (LA) (Hopebio, China) plates with 0.3% (*w*/*v*) agar was used to analyze the swimming motility of different strains. Wild-type strain and its mutant were grown in Luria Broth (LB) broth (Hopebio, China) at 28°C. *E. coli* χ7213 was grown in LB medium supplemented with 50 μg/mL diaminopimelic acid (SCRC, China) at 37°C. CIK cells were cultured at 28°C, 5% CO_2_ in M199 medium (Invitrogen, USA). All mediums contained 10% fetal bovine serum (FBS, Invitrogen, USA) supplemented with 1% penicillin-streptomycin (Invitrogen, USA). Rabbit polyclonal antibody targeting Hcp was produced in our laboratory. Anti-GAPDH polyclonal antibody (Cat # A19056) and HRP goat anti-rabbit IgG (Cat # AS014) were purchased from Abclonal.

**Table 1:** Strains and plasmids used in this study.

**Strains and plasmids**	**Description**	**Source**
**Strains**		
*Aeromonas hydrophila*		
GD18	Wild type	Lab collection
J-1	Wild type	(27)
Δ*vas*H	*vas*H deletion mutant	This work
Δ*hcp*1/2	*hcp*1 and *hcp*2 double-deletion mutant	This work
*Escherichia coli*		
χ7213	*thr*-1 *leu*B6 *fhu*A21 *lac*Y1 *gln*V44 *rec*A1 Δ*asd*A4 Δ(*zhf*-2::Tn10) *thi*-1	(28)
**Plasmids**		
pRE112	Suicide vector, *sac*B, mob^−^(RP4)R6K ori, Cm^r^	(29)
pRE112-*vas*H	pRE112 derivative, designed for knockout of *vas*H, Cm^r^	This work
pRE112-*hcp*1	pRE112 derivative, designed for knockout of *hcp*1, Cm^r^	This work
pRE112-*hcp*2	pRE112 derivative, designed for knockout of *hcp*2, Cm^r^	This work

Healthy grass carp (weighing 200 ± 20 g) were from Xiantao Hatchery (Hubei, China). AB line wild-type zebrafish used in this work were from the Institute of Hydrobiology, Chinese Academy of Sciences (Wuhan, China). Zebrafish were maintained at a density of 10 fish/tank in 8 L tanks. Before infection, fish were acclimatized to the environment for 2 weeks. The animal experiments were performed following animal welfare standards and were approved by the Ethical Committee of Institute of hydrobiology, Chinese Academy of Sciences.

### Genome Sequencing and Assembly

Genomic DNA was extracted from *A. hydrophila* strains GD18 using a TIANamp Bacteria DNA kit (Tiangen, China) according to the manufacturer's instructions. Paired-end (PE) libraries had insert size of 500 bp and 2,000 bp. The sequence of cDNA was generated with an Illumina GA IIx sequencer (Illumina Inc., USA). Sequencing was performed at the Beijing Novogene Technology Co., Ltd. One shotgun run and one 8 kb-library span paired-end run were carried out. De Novo assembly of the raw reads was done by Assembler Software Newbler (version 2.7; Roche/454 Life Science) using default parameters. To obtain clean data, raw reads were processed by removing reads with 5 bp of ambiguous bases, 20 bp of low quality (≤Q20) bases, adapter contamination, and duplicated reads. The final 100× libraries were generated with clean-read data. The reads were assembled with SOAPdenovo v1.05.

### Gene Prediction and Annotation

Putative coding sequences (CDSs) were predicted by Glimmer version 3.0. Transfer RNA (tRNA) genes were explored by the tRNA scan-SE. The rRNAmmer was used to analyze Ribosome RNA (rRNA) genes, while the Rfam database was used to predict small nuclear RNAs (snRNA). Based on the homologous blast method, the transposons were identified using transposon PSI. We used web server PHAST (http://phast.wishartlab.com/) to find prophage sequences and CRISPR Finder.2.3.3 to search for the CRISPR arrays. Functional annotation of CDSs was performed by searching the non-redundant protein database from the NCBI. COGs (clusters of orthologous groups) were obtained from the eggNOG (version 3) database. Proteins with 30% similarity were judged as orthologs and paralogs (30). Metabolic pathways were estimated using Kyoto Encyclopedia of Genes and Genomes (KEGG) database (30). The statistical enrichment of differentially expressed genes in the KEGG pathway was investigated using **KOBAS** software. Genomic islands (GIs) were analyzed by IslandViewer tool. The genome map was drawn by CGView.

### MLST and Phylogenetic Analysis

MLST was performed by amplifying six housekeeping genes (*gyr*B, *gro*L, *glt*A, *met*G, *pps*A and *rec*A) with primers (Supplementary Table 1) as previously described (8). Six housekeeping genes were amplified with primers (Supplementary Table 1). The sequences of distinct alleles were deposited in the *Aeromonas* spp. MLST database (http://pubmlst.org/aeromonas). The STs were determined by the combination of assigned alleles.

The sets of 1,246 concatenated genes used as input for constructing whole cohort phylogenetic trees were generated using OrthoMCL (31). The BLASTp results were transformed into a normalized similarity matrix through OrthoMCL. Markov Cluster algorithm (MCL) was used to cluster orthologous sequences. All of the single-copy homologous proteins and their sequences were extracted from the OrthoMCL clustering results. Multi-sequence alignment of single-copy homologous protein was then sequenced using MAFFT (32). Use Gblocks (Version 0.91b) was used to extract conservative sites of multiple sequence alignment results (33). Maximum likelihood trees were generated with RAxML version 8.0.26 with GTR-GAAMA (34). *Bootstrap* analysis *used* 1,000 *pseudo*-*replicates*. A phylogenetic tree was further visualized using the iTOL tree website (http://itol.embl.de/).

### Construction of *A. hydrophila* Mutants

The mutation of *A. hydrophila* genes was exercised as described previously (35). Primers and plasmids used in this experiment are listed in Table 1 and Supplementary Table 1. The primers were designed based on the whole genome of *A. hydrophila* GD18. The upstream and downstream flanking fragments of *vas*H were amplified with primers P1/2 or P3/4 and were cloned into *Kpn*I sites of pRE112 vector to construct pRE112-Δ*vas*H. We used the donor *E. coli* χ7213 to transfer the suicide plasmids. The mutation was verified by PCR via primers P1/P4. The double-mutant strain Δ*hcp*1/2 was constructed using the same method.

### Hcp Protein Secretion Assay

Western blot analysis was conducted to explore the secretion of Hcp in *A. hydrophila* GD18 and mutant strains as described previously (36). Bacteria were grown in 10 mL LB medium at different temperature conditions, and then centrifuged at 10 000× *g* for 10 min. The cell pellets were resuspended with PBS and supernatants were collected and filtered using a 0.22-μm filter. The samples were separated by 12% SDS-PAGE and transblotted onto *PVDF membrane* (Millipore, USA). The membrane was blocked by 5% non-fat dry milk, then indicated primary antibodies (anti-Hcp at 1:1,000) were used, following secondary antibodies (HRP-conjugated anti-rabbit IgG, 1:5,000). Then, blot bands were visualized with an Image Quant LAS 4,000 system (GE Healthcare, USA).

### The Growth Curve and Virulence Determination

Growth of the Δ*vas*H strain was compared with growth of the wild-type strain GD18 (37). The bacteria were grown in LB medium at 28°C for 8 h with shaking. Then cultures were then inoculated (1:500, v/v) into fresh LB medium. OD_600_ nm reads were taken hourly for 24 h.

The bacterial median lethal doses (LD_50_) were determined in a zebrafish animal infection model as previously described (38, 39). Prior to infection, bacteria were washed in triplicates with sterile PBS and serially diluted. Dilutions were intraperitoneally injected into six groups of zebrafish, 10 fish per group. Negative control zebrafish were injected only with PBS. The fish were observed for 2 weeks and surviving fish were sacrificed on day 14 post-infection. LD_50_ values were determined based on *Karber's* methods (40).

The systemic dissemination capacity of *A. hydrophila* strains were further investigated using grass carp as an infection model. Briefly, grass carp i.p. infected with 10 LD^50^ (2.73 × 10^3^ CFU/fish) by *A. hydrophila* were euthanized and dissected 24 h post-infection. The target organs spleen, kidney, and liver were collected, weighed, and homogenized with PBS. Homogenized samples were plated on LB plates for bacterial count with a ten-fold dilution method.

### Whole Blood Killing and LDH Cytotoxicity Assay

Whole blood killing assay was performed as described by Xie et al. (37). Blood exsanguinated from adult grass carp caudal vein using a sterile syringe with pre-added anticoagulant heparinized following anesthetized with MS-222. 900 μL heparinized blood was mixed with 100 μL bacteria cultures at a concentration of 1 × 10^5^ CFU/mL. The mixtures were then placed at 28°C. 100 μL mixtures were taken at 2 h, serially diluted, spread on LA agar, and incubated at 28°C overnight.

LDH release was assayed using the LDH Cytotoxicity Assay Kit (Promega, USA). CIK cell monolayers were incubated with the GD18 and mutants at a multiplicity of infection (MOI) of 5 for 2 h. The supernatants were collected for measuring the LDH release. The percentage of cytotoxicity was calculated according to the manufacturer's instructions: [(OD_490nm_ sample - OD_490nm_ spontaneous)/ (OD_490nm_ maximum release - OD_490nm_ spontaneous)] ×100. OD_490nm_ spontaneous represented LDH release from uninfected cells into the culture supernatant and OD_490nm_ maximum release indicated LDH release acquired by lysis of the uninfected cells. At least three independent experiments were executed in triplicate wells.

### Bacterial Competition Assays

The bacterial competition assay was carried out as previously described with minor modifications (41). *E. coli* DH5α was transformed with pET-28a to confer kanamycin resistance. All bacteria strains were grown to the logarithmic phase (OD_600nm_ of 0.5). The attacker *A. hydrophila* (ampicillin-resistant) and the prey *E. coli* DH5α (kanamycin-resistant) were mixed at a ratio of 1:5. The mixture was incubated on LA plates with a nitrocellulose membrane at 28°C for 3 h. Surviving *E. coli* were collected and serially diluted onto kanamycin LB plates, then incubated for 24 h. Each assay was performed three independent times in triplicate.

### Analysis of T6SS Core Genes Expression Levels by qRT-PCR

Gene expression of T6SS core genes was measured by qRT-PCR (42). *A. hydrophila* strains were incubated in LB medium or LB medium with 50% grass carp serum until mid-log phase (OD_600__nm_ of 0.6) and used for RNA-extraction. Total RNA was isolated using Trizol reagent (Invitrogen, USA). Reverse transcription was carried out using M-MLV reverse system (Promega, USA) and the random primers following the manufacturer's instructions. Quantitative real-time PCR (qPCR) using Fast SYBR Green PCR Master Mix (Bio-Rad, USA) was run on the CFX96 Real-Time System (Bio-Rad, USA). All primers used for qPCR are shown in Supplementary Table 1. Gene expression was calculated according to the 2^−ΔΔCT^ method. The 16sRNA gene was used as a reference gene for normalization. The relative expression level was obtained as the ratio compared to that of the wild-type strain GD18. All *experiments were* independently conducted three times.

### Statistical Analysis

Prism GraphPad 8 (GraphPad Software) was employed for statistical analysis. Unpaired *t*-tests and two-way ANOVA followed by multiple comparisons were used for statistical analysis. A *p*-value of < 0.05 was significant statistically (^*^p < 0.05, ^**^p < 0.01, ^***^p < 0.001, ^****^p < 0.0001). *All* the *experiments were repeated* thrice independently before analyzing data.

## Results

### Biological Characteristics and Multilocus Sequence Type Analysis of Virulent *A. hydrophila* GD18

**Figure 1:**
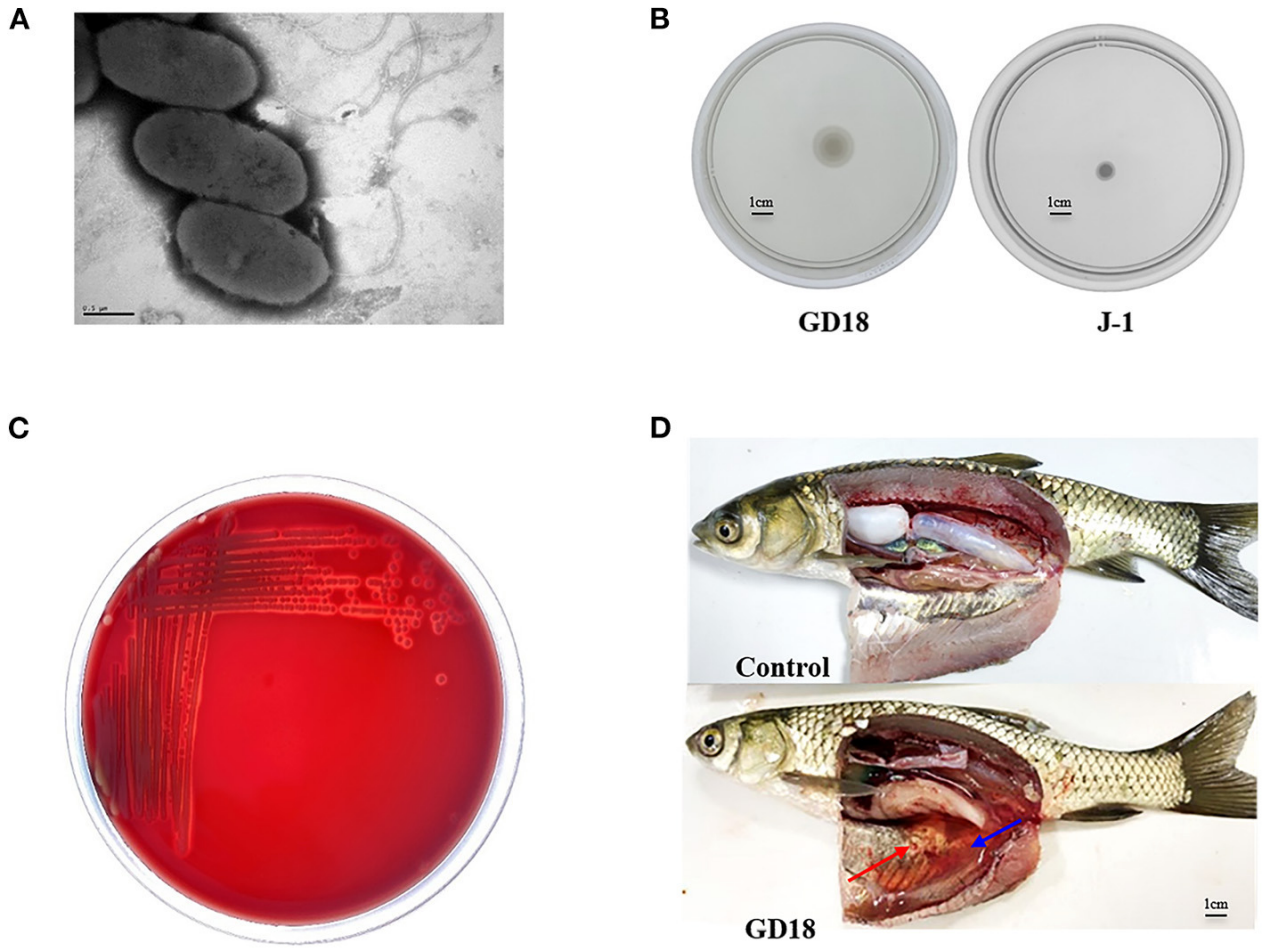
Biological characteristics of *A. hydrophila* GD18. **(A)** Morphological characteristics of *A. hydrophila* GD18 observed under a transmission electron microscopy. **(B)** Swimming motility of *A. hydrophila* GD18. **(C)** The hemolytic activity of *A. hydrophila* GD18 was confirmed by plating on a blood agar plate. **(D)** Grass carp i.p. infected with 2.73 × 10^3^ CFU/fish by *A. hydrophila* GD18 displayed typical symptoms of motile *Aeromonas* septicemia after 24 h. The blue arrow indicates massive ascites and the red arrow indicates the peritoneal mucosa bleeding spots.

*A. hydrophila* GD18 was isolated from sick grass carp (*C. idella*) in Guangdong province, China, in 2017. Observed by transmission electron microscopy, *A. hydrophila* GD18 is rod-shaped and possesses one polar flagellum (Figure 1A). Consistently, it could spread on a swimming agar plate (Figure 1B). Typical β-hemolysis was also detected, indicating *A. hydrophila* GD18 could produce and secrete β-hemolysins (Figure 1C). The grass carp intraperitoneally (*i.p*.) infected by *A. hydrophila* GD18 showed the same symptoms as the naturally infected fish, such as diffuse hemorrhages on the skin and abdominal swelling. After dissection, massive ascites flowed out, and internal organs exhibited hyperemia (Figure 1D). Zebrafish is a model animal for measuring the virulence of *A. hydrophila* (43). The LD_50_ of GD18 in zebrafish was 2.73 × 10^2^ CFU/fish, which was indicative of its high pathogenicity to fish.

To determine the epidemiological relation of *A. hydrophila* GD18 with other isolates, MLST was performed. The concatenated sequences of the six alleles (*gyr*B, *gro*L, *glt*A, *met*G, *pps*A, and *rec*A) of GD18 were different from the ST251 group, which is considered to be accountable for the ongoing MAS outbreaks in China and the Southeastern United States. GD18 was found to belong to a new ST656, which hasn't been reported so far (see Table 2).

**Table 2:** The multilocus sequence typing (MLST) of *A. hydrophila*.

**Strains**	**Host**	**Country**	**Year**	**ST**	**Allele**					
					***gyr*B**	***gro*L**	***glt*A**	***met*G**	***pps*A**	***rec*A**
GD18	Grass carp	Guangdong, China	2017	**656**	415	164	160	267	457	160
J-1	Crucian carp	Jiangsu, China	1989	**251**	210	214	122	211	221	217

*To emphasize that this is the result of the ST sequence type*.

### Genome Sequencing and Phylogenetic Analysis

**Figure 2:**
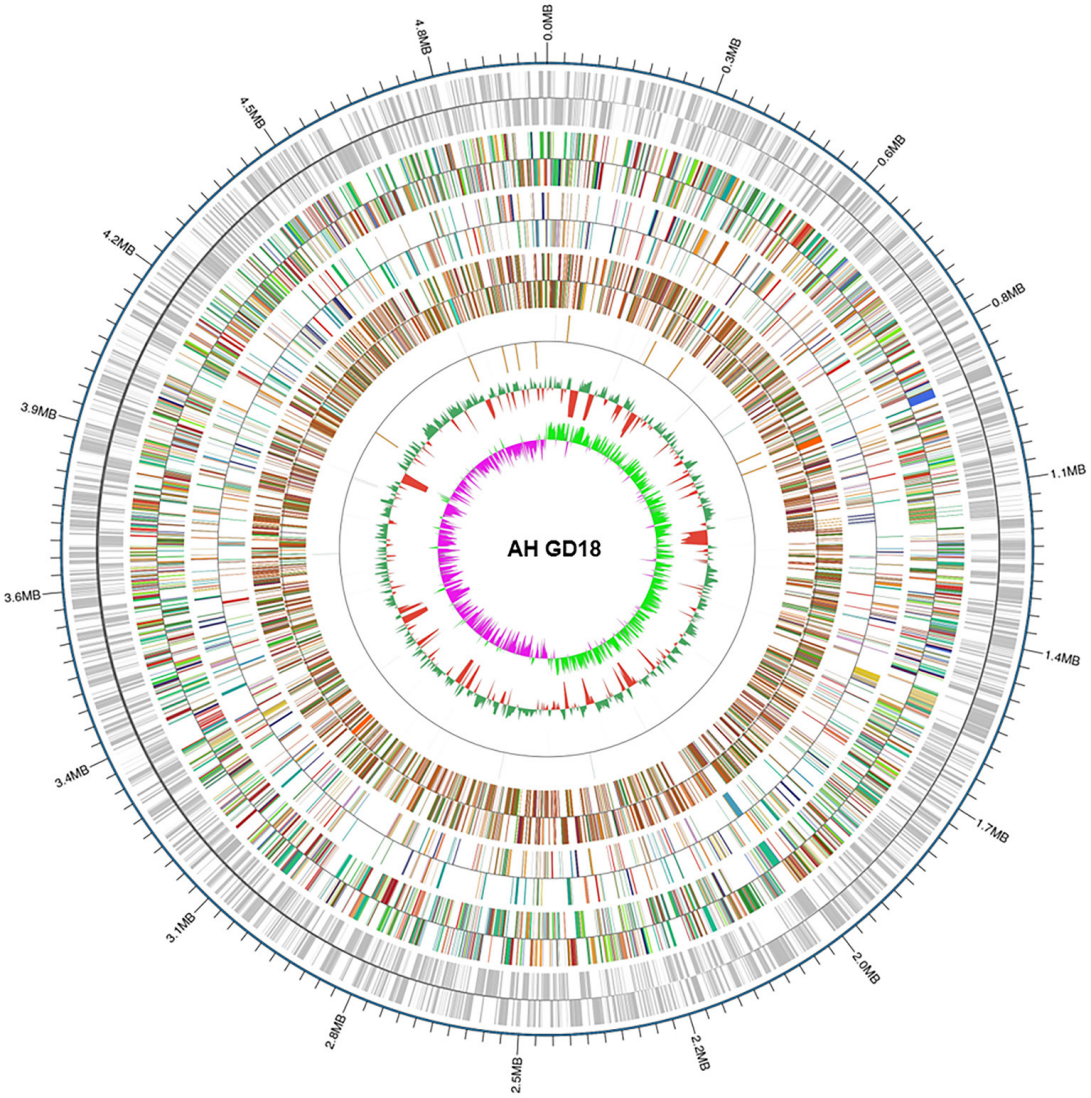
Genome map of *A. hydrophila* GD18. Circular map for the whole genome of *A. hydrophila* GD18. From the outside to the center: genome sequence coordinates, gene annotation (COG, eggNOG, KEGG, and GO categories), ncRNA, GC content, and GC skew (G – C/G + C).

Considering the new ST of *A. hydrophila* GD18, whole-genome sequencing was applied. The genome size is 4,946,275 bp with 61.03% GC content (Figure 2). A total of 126 non-coding RNAs were also predicted in the GD18 genome. The analysis showed that there were no plasmids. CRISPR is a specific family of DNA direct repeat sequences that is broadly distributed in prokaryotic genomes. One CRISPR array was predicted in the GD18 genome.

**Figure 3:**
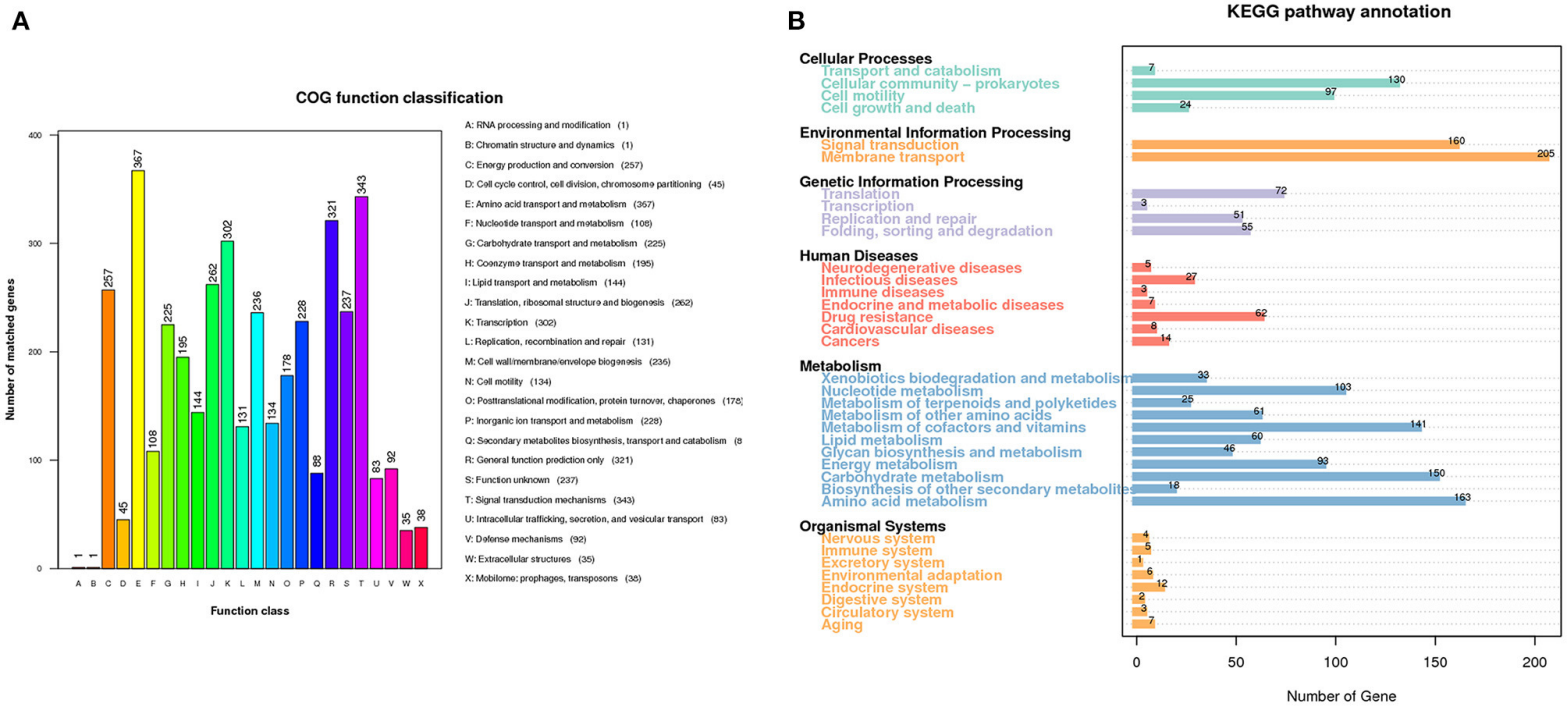
Gene functional analysis of *A. hydrophila* GD18. **(A)** Gene functional classifications of *A. hydrophila* GD18 based on the Clusters of Orthologous Groups (COG) proteins database. **(B)** Metabolic pathways categories of *A. hydrophila* GD18 genes based on the KEGG database.

A total of 4,571 open reading frames (ORFs) were found with an average length of 904 bp, constituting 83.57% of the genome. 4,051 out of the 4,571 ORFs were annotated into 24 categories in the COG database (Figure 3A). The six most abundant categories were amino acid transport and metabolism (367), signal transduction mechanisms (343), transcription (302), translation, ribosomal structure and biogenesis (262), energy production and conversion (257), and cell wall/membrane/envelope biogenesis (236). The numbers of genes annotated in the RNA processing and modification (1), and chromatin structure and dynamics (1) categories are the least.

KEGG database is a collection of the molecular interaction and reaction networks in cells and organisms. 1,863 out of the 4,571 ORFs were annotated into 36 biological pathways of six superfamilies in the KEGG database (Figure 3B). Consistent with those derived from the COG database, the metabolism superfamily was the most abundant with total of 894 genes. Notably, the second most abundant was the environmental information processing superfamily, with 160 genes in the signal transduction pathway and 205 genes in the membrane transport pathway, coincident with its ecological adaption. Furthermore, 27 and 62 genes were annotated to have the functions of infectious diseases and drug resistance, respectively.

**Figure 4:**
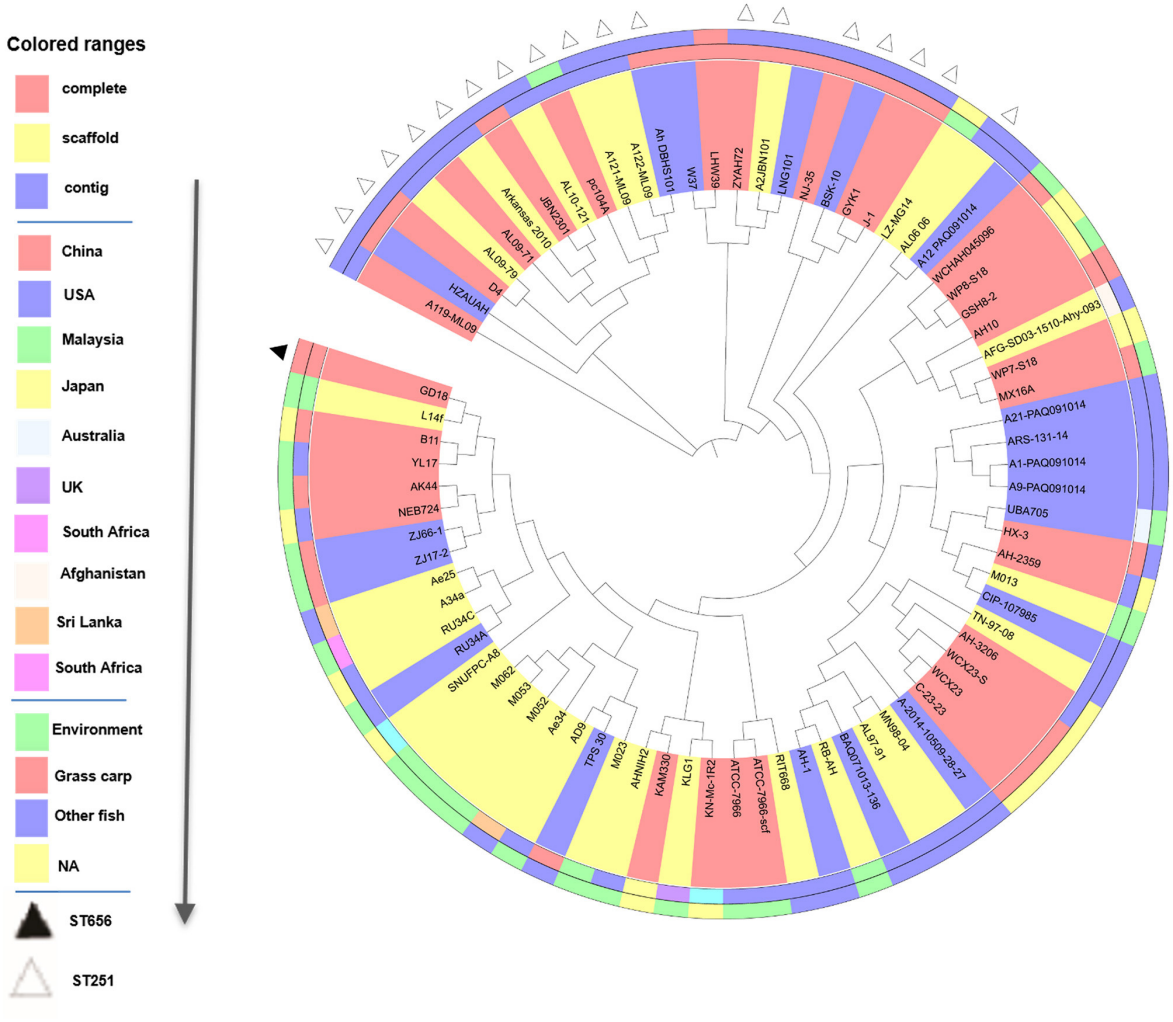
Phylogenetic tree analysis of 78 *A. hydrophila* strains. Phylogenetic tree based on the 1246 core genes of the 78 *A. hydrophila* isolates. Sequencing type, isolation country, and source of *A. hydrophila* strains are color-coded in the following rings. On the outermost ring, STs are distinguished by triangles.

We then performed phylogenetic analysis to investigate the evolutionary relationship of GD18 with other *A. hydrophila* isolates. The phylogeny tree was built with 78 fish and environmental *A. hydrophila* strains based on the 1,246 core genes (Figure 4). Strain L14f, isolated from a lake water sample in Malaysia, was found in close proximity to GD18. Most of the strains in the clade to which the GD18 strain belongs are environmental isolates. The epidemic strains including Chinese isolates NJ-35, J-1, and American isolates ML09-119, AL09-71 and pc104A formed separate linages, and fell into nearby clades. The ST251 strains also clustered closely but distantly related to ST656 strain GD18. Based on this phylogenetic analysis, GD18 was closer to environmental isolates, including the *A. hydrophila* reference strain ATCC 7966^T^ than to epidemic strains. Hence, the mechanism of how *A. hydrophila* GD18 balances the environmental adaptability with virulence is worth further study.

### T6SS in *A. hydrophila* GD18 and the Regulation Condition

**Figure 5:**
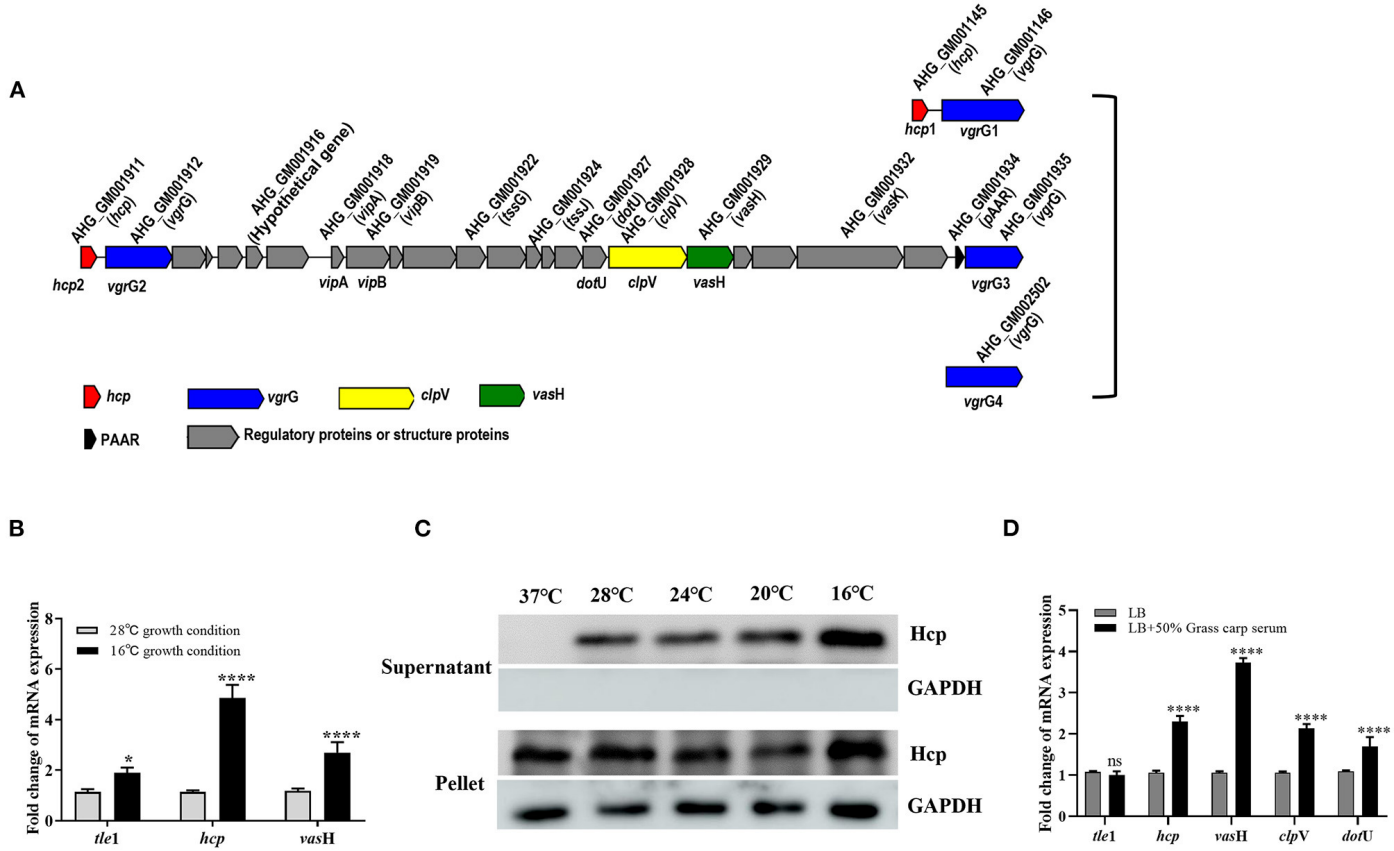
T6SS is conditionally regulated in *A. hydrophila* GD18. **(A)** Genetic organization of the T6SS major structural gene cluster in *A. hydrophila* GD18. **(B)** Transcripts of the T6SS genes are elevated at 16°C comparing to that at 28°C. **(C)** Expression and secretion of Hcp protein at different temperatures. Anti-GAPDH antibody served as an internal reference. **(D)** Grass carp serum significantly upregulated transcripts of T6SS core genes at 28°C. The transcripts in the indicated conditions were analyzed by qRT-PCR. In B and D, *A. hydrophila* cultures were grown until mid-log phase (OD600 _nm_ of 0.6) and used for RNA-extraction. The data was presented as the mean ± SD of three independent experiments. In C, the data was from one representative experiment with at least three independent biological replicates. Statistical significance was calculated by 2-way ANOVA followed by Sidak's multiple comparisons test. ns, not significant; *p < 0.05; ****p < 0.0001.

Bacterial cells communicate with their surroundings via the secretory system. As one of the most recently identified secretion systems, T6SS can convey toxins into eukaryotic cells as well as other bacteria, highlighting the importance of the T6SS not only in the context of infection and disease but for efficient competition with indigenous microbiota for limited resources (17). The T6SS gene cluster of GD18 was found to cover 21 kb with 25 conserved T6SS core genes from AHG_GM1911 to _GM1935 (Figure 5A). AHG_GM1911, named *hcp*2, encoded the ortholog of the Hcp superfamily. There were two VgrG superfamily genes in the T6SS gene cluster. AHG_GM1912 was named *vgr*G2 and AHG_GM1935 was named *vgr*G3. AHG_GM1934 encoded a PAAR-repeat protein which assembles a sharp appendix on the VgrG tip. AHG_GM1918 named *vip*A, and AHG _GM1919 named *vip*B, formed a polymerization sheath structure surrounding the tube rings. AHG_GM1928 belonged to the ClpV1 superfamily and was named *clp*V, dissociating the VipA/VipB complex to power the T6SS. The gene encoding the other Hcp (named *hcp*1) and the two genes encoding VgrG (named *vgr*G1 and *vgr*G4) were also found outside the T6SS cluster. The secretion of Hcp is thought to be a reliable marker of functional T6SS (44). When GD18 grew to 6 h, Hcp was detectable in both whole-cell and supernatant samples, suggesting that T6SS of GD18 is functional (Supplementary Figure 1).

Conditional expression of T6SS is thought to be favorable for the survival of bacteria in the natural habitat and interaction with their hosts. The regulation condition of T6SS in GD18 was investigated. We first compared the transcriptional levels of the T6SS genes at 28°C with that at 16°C. The transcriptional levels of *tle*1 (45), *hcp* and *vas*H exhibited 1.9-fold (*p* < 0.05), 4.7-fold (*p* < 0.0001) and 2.7-fold (*p* < 0.0001) increase, respectively, at 16°C compared to the 28°C culture conditions (Figure 5B). In consistence, the expression of the Hcp protein increased with temperature decreasing in both whole-cell and culture supernatant samples (Figure 5C). The Hcp could not be detected in the supernatant when the temperature was raised to 37°C, indicating the inactivation of T6SS at this temperature.

It has been proved that the T6SS expression of *Pseudomonas aeruginosa, Salmonella* Typhimurium, and avian pathogenic *Escherichia coli* was increased during infection (42, 44–46). Considering the bactericidal property, fish serum was used to simulate *in vivo* conditions. It was revealed that the transcripts of all the T6SS core genes, including *hcp, vas*H, *clp*V, and *dot*U, increased under grass carp serum conditions relative to LB conditions. In particular, the highest up-regulated gene was *vas*H (3.7-fold) (*p* < 0.0001) (Figure 5D). In summary, T6SS is conditionally regulated in GD18.

### VasH Contributes to the T6SS-mediated Bactericidal Activity of *A. hydrophila* GD18

**Figure 6:**
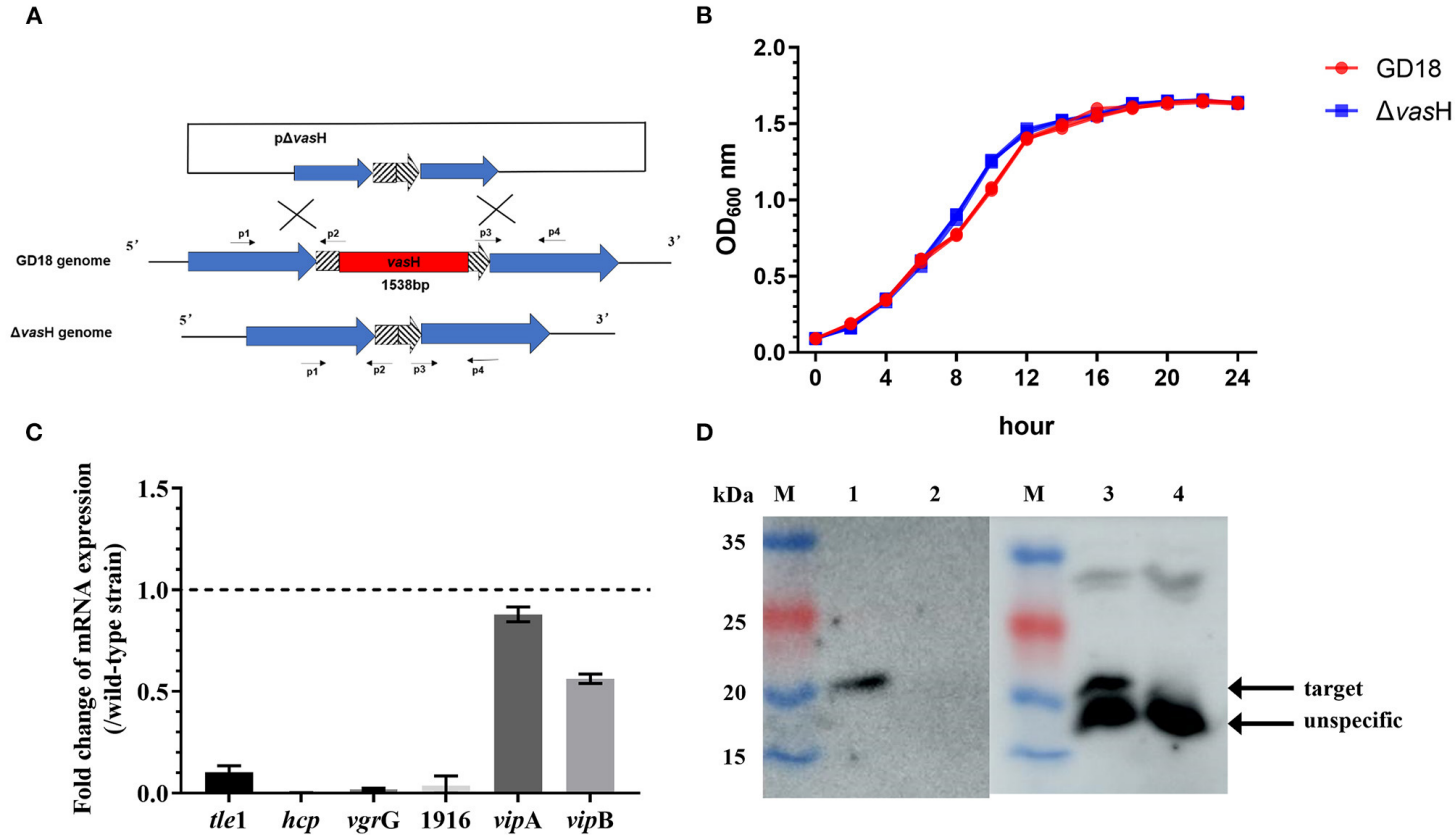
Construction and phenotypic characterization of Δ*vas*H strain. **(A)** Construction strategy of Δ*vas*H by homologous recombination. **(B)** Growth curves in LB medium over a 24 h period at 28°C. **(C)** Fold change of transcriptional levels of the T6SS genes in Δ *vas*H comparing to that in the wild-type strain at 28°C. The mRNA level of each gene was normalized to that of 16S rRNA. The data was presented as the mean ± SD of three independent experiments. **(D)** Hcp expression and secretion in whole-cell and supernatants of wild-type and mutant strains. Lane M, PageRuler prestained protein ladder; lane 1, GD18 supernatant; lane 2, Δ*vas*H supernatant; lane 3, GD18 whole-cell; lane 4, Δ*vas*H whole-cell. The data was from one representative experiment with at least three independent biological replicates.

Transcription of *hcp* is regulated by a multiple bacterial enhancer binding protein (bEBP) VasH in *V. cholera* (47). In *A. hydrophila* GD18, the large T6SS gene cluster contains gene *vas*H. To explore the function of VasH in GD18, a *vas*H deletion mutant (Δ*vas*H) was constructed by homologous recombination (Figure 6A). Δ*vas*H mutant has a similar growth rate with **wild-type** strain in culture condition (Figure 6B).

Deletion of the *vas*H gene totally abolished the transcription of *hcp* and the expression of Hcp, indicating the inactivation of T6SS (Figures 6C,D). In addition, the transcription of T6SS core genes, including *vgr*G, AHG_GM1916 (hypothetical protein-coding gene), antibacterial effector *tle*1 significantly decreased in Δ*vas*H than in the wild-type strain (Figure 6C). On the contrary, the impact of *vas*H mutation on *vip*A and *vip*B transcripts was relatively limited.

**Figure 7:**
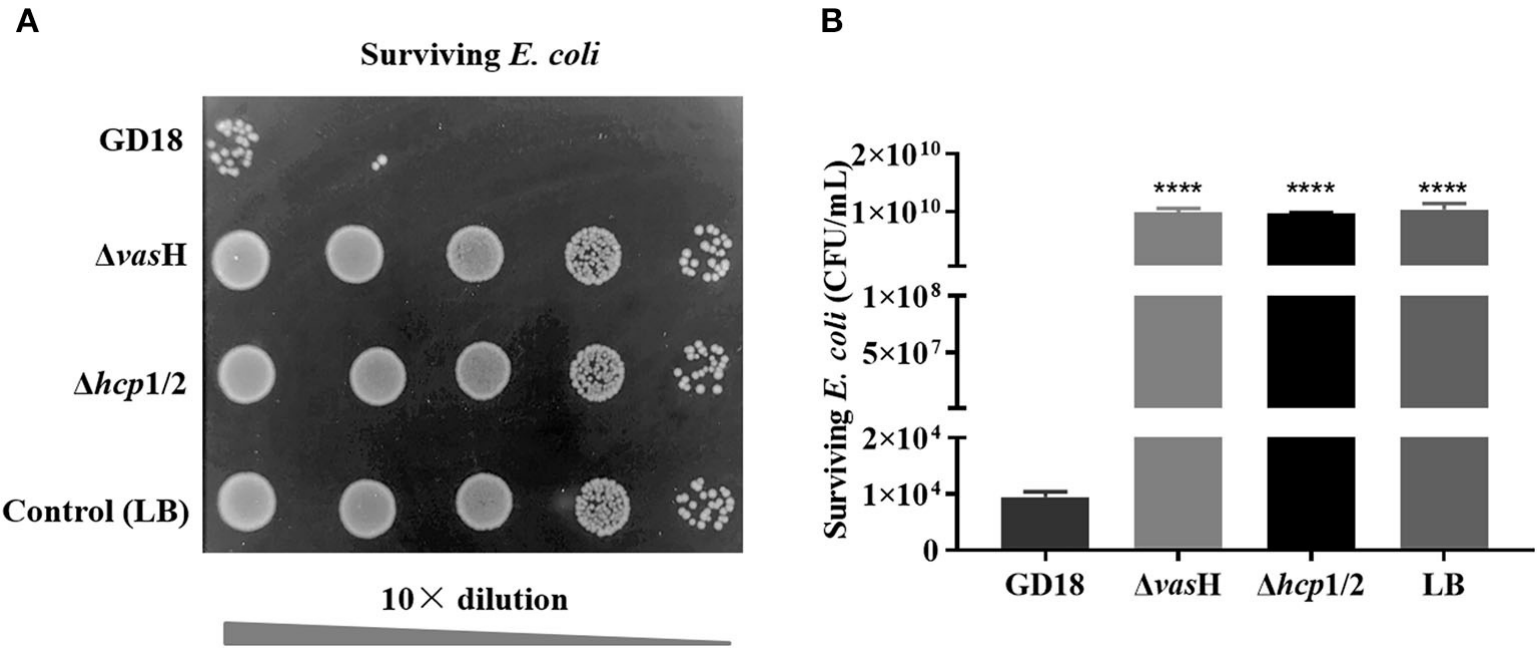
VasH contributes to the bactericidal effect of *A. hydrophila*. The survival of *Escherichia coli* was determined post-incubation with different *A. hydrophila* strains as indicated. **(A)** The surviving *E. coli* cells were serially diluted and determined on the LA plates supplemented with kanamycin. Control indicates incubation of *E. coli* with sterile LB medium alone. Δ*hcp*1/2 mutant group was set as the T6SS defective control group. **(B)** The data was presented as the mean ± SD of three independent experiments. Statistical significance was calculated by unpaired *t*- test. ****p < 0.0001.

To determine whether VasH contributed to the bactericidal activity of *A. hydrophila* GD18, growth competition experiments were conducted (Figure 7). When co-cultured with *A. hydrophila* GD18, the survived *E. coli* reduced six log_10_ in number compared to *E. coli* cultured alone. Compared with the wild-type strain group, the inhibition of *E. coli* growth by Δ*vas*H was markedly reduced. Δ*hcp*1/2 was set as a positive control. These results suggested that the T6SS is vital to the antibacterial activity of *A. hydrophila* GD18 and that VasH takes part in T6SS regulation and mediating the bactericidal activity.

### VasH Contributes to Cytotoxicity and Resistance Against Fish Blood Killing

**Figure 8:**
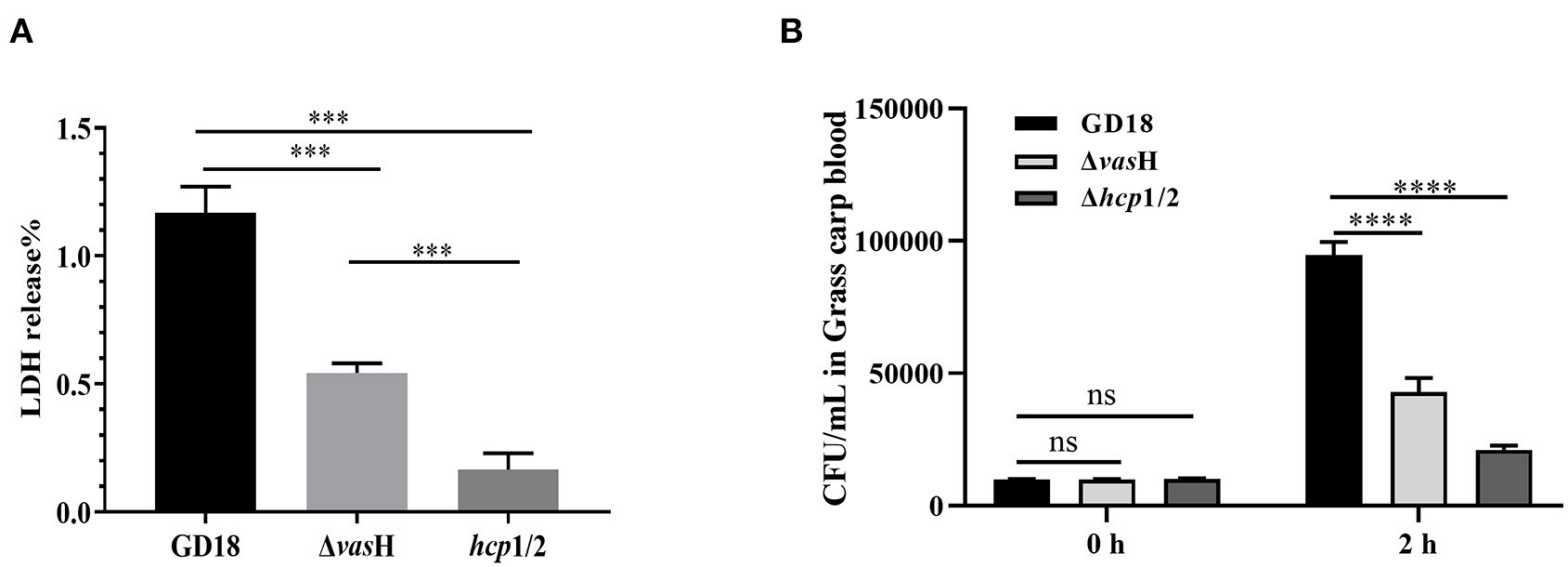
VasH contributes to cytotoxicity and resistance against fish blood killing. **(A)** Cytotoxicity results after 2 h of incubation. **(B)** Bacteira number following 2 h of incubation with whole blood of grass carp. The data was presented as the mean ± SD of three independent experiments. In A, statistical significance was calculated by unpaired t- test. In B, statistical significance was calculated by 2-way ANOVA followed by Sidak's multiple comparisons test. ns, not significant; ***p < 0.001, ****p < 0.0001.

The cytotoxic effect of *A. hydrophila* strains was tested by determining the activity of the LDH enzyme of CIK cells. Compared to the wild-type strain, Δ*vas*H infection caused a significant decreased (53%) (*p* < 0.001) of LDH release by CIK cells after 2 h of infection at a MOI of 5 (Figure 8A).

Furthermore, we explored the resistance against host killing of *A. hydrophila* strains in grass carp blood. Both of the Δ*vas*H and wild-type strains proliferate after incubation with heparinized fish blood, suggesting whole blood cannot effectively kill both Δ*vas*H and wild-type strains. However, after 2 h of incubation, the bacteria number of Δ*vas*H was 4.29 × 10^4^ CFU/mL and that of wild-type strain was 9.46 × 10^4^ CFU/mL, demonstrating Δ*vas*H was less resistant to the bactericidal effect of grass carp blood (*p* < 0.0001) (Figure 8B).

### VasH Is Required for Virulence and Systemic Dissemination of *A. hydrophila* GD18

**Figure 9:**
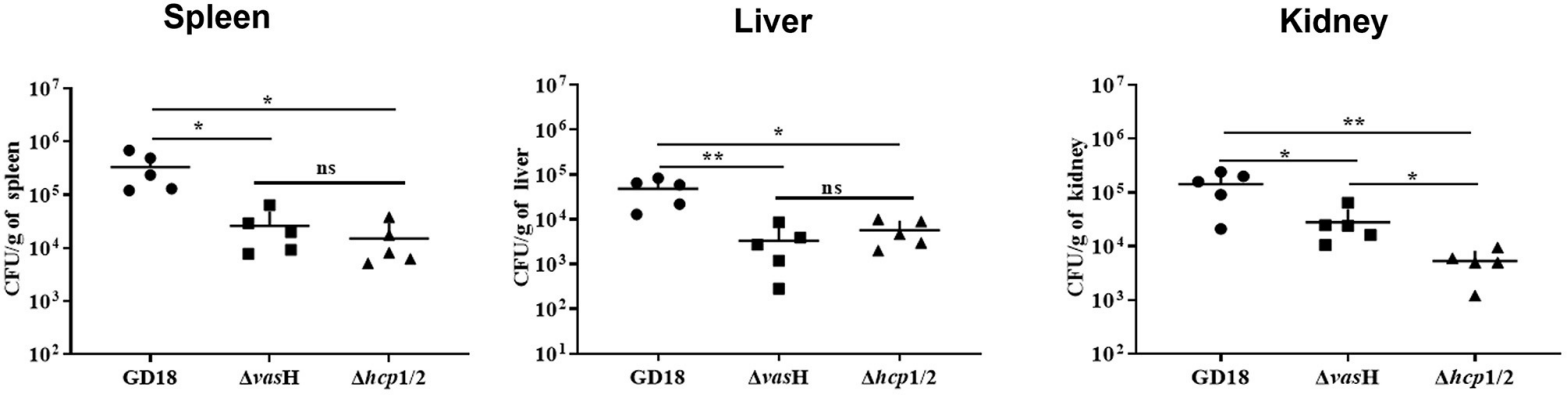
VasH is required for systemic dissemination of *A. hydrophila* GD18. Grass carp i.p. infected with 2.73 × 10^3^ CFU/fish by *A. hydrophila* were euthanized and dissected 24 h post-infection. The data was presented as mean ± SD of five biological replicates from one representative experiment of three independent experiments. Statistical significance was calculated by unpaired *t*- test. ns, not significant; *p < 0.05; **p < 0.01.

To determine whether the mutation of *vas*H affects virulence, we further calculated the LD_50_ values of different strains using a zebrafish intraperitoneally infection model. The LD_50_ value of *A.hydrophila* GD18 was 2.73 × 10^2^ CFU (see Table 3), while Δ*vas*H had a nearly 4.4-fold higher LD_50_ value. The results indicated that VasH contributes to the virulence of *A. hydrophila* GD18. Moreover, the deletion of the *vas*H decreased capacity of systemic dissemination. The bacterial loads of the Δ*vas*H in the organs, including spleen, kidney, and liver were reduced by 42, 93, and 80%, respectively, comparing to those of wild-type (Figure 9).

**Table 3:** Calculations of LD_50_s of the *A. hydrophila* GD18 and mutant strains in zebrafish.

**Dose of challenge CFU**	**Number of death/Total**			**Survival rate (%)**		
	**GD18**	**Δ*vas*H**	**Δ*hcp*1/2**	**GD18**	**Δ*vas*H**	**Δ*hcp*1/2**
10^5^	10/10	10/10	10/10	0	0	0
10^4^	10/10	8/10	5/10	0	20	50
10^3^	9/10	5/10	2/10	10	50	80
10^2^	0/10	0/10	0/10	100	100	100
LD_50_^*^	2.73 × 10^2^	1.19 × 10^3^	5.62 × 10^3^			

* *The LD_50_ was calculated according to Karber's method*.

## Discussion

*Aeromonas hydrophila* is ubiquitous in various aquatic environments and causes disease in fish, reptiles, amphibians, and humans (48). This organism has evolved a variety of successful apparatus under competitive forces to adapt to various habitats both *in vitro* and *in vivo*. In this study, *A. hydrophila* GD18 was isolated from sick grass carp. Because of the high yield and desirable flavor, grass carp is one of the most dominant freshwater fish species in China. Nevertheless, the decline of the aquaculture environment and germplasm degradation of grass carp species lead to increasing *A. hydrophila* infection, resulted in heightened economic losses, which raised more and more attention (49).

Observation with the transmission electron microscope clearly showed that *A. hydrophila* GD18 possesses polar flagellum. And the swimming motility halo of GD18 tested by the swimming plate showed a distinct motile phenotype with a large diffuse spreading diameter. It is known that polar flagella are usually important locomotive organelles and virulence factors for bacterial motility and colonization (50). Motile aeromonad is the causative agent of MAS in fish (51). Motility takes a leading role in the initial phases of the infection in bacterial pathogens (50). The above phenotype and the presence of β-hemolysins alluded to the pathogenic capabilities of GD18. The grass carp intraperitoneally infected by GD18 also exhibited clinical signs typical of hemorrhagic septicemia. Furthermore, the LD_50_ of GD18 in zebrafish (2.73 × 10^2^ CFU/fish) was much less than the “virulence” criteria (<1.0 × 10^6^ CFU/fish) used by Pang et al. (9), indicating that GD18 could be classified as a high virulent strain.

Accompanied with confusion and argument, the taxonomy of the genus *Aeromonas* is complicated (48). MLST is an explicit method to identify the bacterial isolates based on the allelic profiles of house-keeping genes (48). The previous study concluded that ST251 is a risky group and would be responsible for the MAS outbreaks in recent years, as 17 virulent strains including the five epidemic strains all belonged to ST251 (9). Moreover, there was a high relevance between the genetic phylogeny and pathogenicity. The strains that belonged to ST251 clonal group all exhibited virulence while the other ST strains were avirulent in zebrafish (9). *A. hydrophila* GD18 was determined to belong to a novel serotype of ST656, which hasn't been described in any published literature. Considering its pathogenic potential verified in this study, complete genome sequencing was carried out to provide a comprehensive understanding of this strain.

According to COG and KEGG-based functionally annotation, the core genome was enriched in metabolism-related genes, followed by environmental information processing superfamily like signal transduction pathway. To note, both COG and KEGG databases produced consistent results, explaining the relationship between the gene function and environmental adaption mechanism of *A. hydrophila* GD18. Previously, the comparative genome analysis of 49 *A. hydrophila* genomes revealed that core genes were higher among the classes of substance dependence, amino acid metabolism, cell cycle and endocrine system, yet not mentioned genes related to environmental information processing and environmental adaptation (52). These genomic features reflect the evolutionary adaptation of *A. hydrophila* strains to different environments and infection strategies.

To determine the evolutionary relationships of GD18 with other *A. hydrophila* strains, 78 *A. hydrophila* genome sequences (31 complete, 28 scaffold, and 19 contig genomes) were obtained from the NCBI database. Phylogenic analysis based on core-genome demonstrated that GD18 clustered in one branch with strain L14f, B11, YL17, AK44, NEB724, ZJ66-1, and ZJ17-2. Five of these strains were environmental isolates. The branch was distantly away from the ST251 clone. According to available literature, L14f was isolated from lake water, B11 was isolated from diseased *Anguilla japonica* (53), and YL17 was isolated from a compost pile (53). Among them, B11 was the only know virulence strain, with a LD_50_ of 2.98 × 10^4^ CFU/ml (1.49 × 10^3^ CFU/fish) in zebrafish (53). The pathogenicity of the other isolates has not been reported so far. In the previous studies of *V. cholera*, environmental strains were considered a repository of virulence genes (54–56). The possibility for “mixing and matching” of genes in the environment pool resulting in new pathogenic variants should be taken more seriously. Similarly, *A. hydrophila* exists in aquatic ecosystems as an inherent resident globally. Further studies on the ecology and evolution of *A. hydrophila* will undoubtedly provide valuable perceptions into the epidemiology of MAS. For strain GD18, the close evolutionary relationship with environmental strains and pathogenic characteristics make it particularly important to uncover the mechanism of balancing the relationship between the two aspects.

T6SS is vital in interbacterial competition and is a major virulence determinant for numerous Gram-negative bacteria. T6SS translocates effectors into the extracellular surroundings, and frequently into neighboring prokaryotic or eukaryotic cells (17). Our study discovered that *A. hydrophila* GD18 gemome has a complete T6SS cluster. Further detection of Hcp in the culture supernatants confirmed the T6SS was functional. For allowing the bacteria to thrive in a competitive environment and to occupy the niche successfully, the expression of T6SS is tightly regulated. In *Yersinia pestis* and *V. cholerae* O1 strains, the T6SS gene cluster has been shown to be induced at low temperature rather than host body temperature. Along these lines, the activation of T6SS was regarded to assist the environmental survival and infection (56). In *A. hydrophila* GD18, Hcp and VasH expression was strongly induced by low temperature (16°C), suggesting that the activation of T6SS promotes the *A. hydrophila* environmental survival. To note, the secretion of Hcp was totally abrogated at 37°C. As *A. hydrophila* GD18 was fish-isolated, the pathogenicity to warm-blooded animals was still unknown. Fernández-Bravo et al. presented a human case of necrotizing fasciitis due to co-infection with 4 *A. hydrophila* strains (NF1–NF4). NF1 strain was determined to be phylogenetically distinct and exhibited contact-dependent killing of NF2 mediated by T6SS at 37°C (57). Therefore, different *A. hydrophila* isolates may employ particular temperature-regulation mechanisms of T6SS to adapt to different environments and hosts.

The importance of T6SS in pathogenesis is becoming increasingly evident. The known genes related to T6SS integration have also been shown to contribute to the virulence of *Aeromonas* (58, 59), *Salmonella* (60), *Fracisella* (61), and *Edwardsiella* (62). Consistent enhancement of transcripts of T6SS genes under grass carp serum conditions suggests an essential role for the T6SS in *A. hydrophila* infection. Among them, *vas*H, a σ^54^-transcriptional activator coding-gene, with the highest upregulation attracted our attention. To assess the function of VasH in *A. hydrophila*, a *vas*H mutant was constructed. It showed that the secretion and expression of Hcp were abolished in Δ*vas*H. In addition, the transcription of T6SS core genes *vgr*G, AHG_GM1916, and *tle*1 all decreased in Δ*vas*H than in the wild-type strain. In *V. cholera* O1, VasH is necessary for the functional T6SS as it regulated Hcp production (63). Suarez et al. provided evidence that *vas*H was necessary for the expression of Hcp in clinical *A. hydrophila* isolate SSU, which was later reclassified as *A. dhakensis* (7, 23). In *V. fischeri*, it was shown that σ54 interacts with RNA polymerase at the promoter region of *hcp*. Meanwhile it was proposed that hexameric VasH binds to the upstream of promoter (26). Results in this study indicated that VasH in *A. hydrophila* GD18 involved in the transcription regulation of not only T6SS apparatus protein but also anti-bacterial effector protein such as Tle1 (45). Future investigations are required to determine the regulation mechanism.

Pathogens using T6SS as an anti-microbial weapon can effectively compete with the natural microflora for limited resources (46). Therefore, we sought to determine whether VasH-mediated T6SS regulation provides competition and pathogenesis fitness to *A. hydrophila* GD18. The mutation of *vas*H significantly reduced the antibacterial activity, similar with the T6SS defective mutant Δ*hcp*1/2. The affected bactericidal capacity in Δ*vas*H could attribute to the failure of producing and assembling a functionally T6SS structure or decreased transcription of the antibacterial effectors. Wang et al. illustrated that T6SS of *A. hydrophila* contributes to the survival and infection (35). Our results supported that the disruption of the *A. hydrophila* T6SS and VasH resulted in defective anti-host killing, cytotoxicity, diminished systemic dissemination ability, and attenuated virulence in grass carp.

*A. hydrophila* is a ubiquitous organism in aquatic environments and also an important opportunistic pathogen. The mechanism of this waterborne pathogen to balance the environmental persistence and outbreak potential is intriguing. In this study, we reported the complete genome of a fish-pathogenic *A. hydrophila* strain GD18, which belongs to a new sequence type ST656. GD18 was found to be closely related to environmental isolates but showed high pathogenicity to fish hosts. The further analysis supported that T6SS greatly contributed to the bactericidal activity and pathogenicity and was regulated by the bacterial enhancer-binding protein VasH. The high-quality whole-genome sequences generated in this study laid an essential foundation for future studies. Moreover, investigation of the VasH would provide valuable perception into the regulation of T6SS and exciting candidates for an attractive target of therapeutics, vaccine, and antimicrobial drug development against *A. hydrophila*.

## Data Availability Statement

The data presented in the study are deposited in the Dryad Digital repository. Please refer to the following link: https://datadryad.org/stash/share/YHITTMMGU86518rOZ1KxNEVtE04nDhDUfUztyW2owZo.

## Ethics Statement

The animal study was reviewed and approved by the Ethical Committee of Institute of hydrobiology, Chinese Academy of Sciences.

## Author Contributions

Y-AZ and YZ: conceived and designed the experiments and writing—review and editing. JL and ZW: performed the experiments. JL, ZW, CW, and D-DC: data curation. JL: writing—original draft. All authors have read and agreed to the published version of the manuscript.

## Funding

The study was supported by the National Natural Science Foundation of China (31772889, 32073022, and 32002431) and China Agriculture Research System (CARS-46).

## Conflict of Interest

The authors declare that the research was conducted in the absence of any commercial or financial relationships that could be construed as a potential conflict of interest.

## Publisher's Note

All claims expressed in this article are solely those of the authors and do not necessarily represent those of their affiliated organizations, or those of the publisher, the editors and the reviewers. Any product that may be evaluated in this article, or claim that may be made by its manufacturer, is not guaranteed or endorsed by the publisher.
