# Supplementary material for: VasH Contributes to Virulence of Aeromonas hydrophila and Is Necessary to the T6SS-mediated Bactericidal Effect
Source: Front Vet Sci. 2021 Dec 13;8:793458. doi: 10.3389/fvets.2021.793458 (PMC8710571; doi:10.3389/fvets.2021.793458)
Supplement: Supplementary file 2 [file Data_Sheet_1.docx]

## Supplementary Figures

**Fig. S1 Hcp expression in whole cell and secretion in supernatant of *A. hydrophila* GD18.**
